# Supplementary material for: Impact of Social and Economic Determinants on the Prevalence of Childhood Overweight and Obesity: A Cross-Sectional Study from the ENPIV in Valencia, Spain
Source: Nutrients. 2025 Jun 15;17(12):2006. doi: 10.3390/nu17122006 (PMC12195892; doi:10.3390/nu17122006)
Supplement: Supplementary file 1 [file nutrients-17-02006-s001.zip › ENPIV_Supplementary_Material_clean.pdf]

Supplementary Materials

STROBE Statement

Table S1. STROBE Checklist for Cross-Sectional Studies report, with section and line references.

	Item No	Recommendation	Location in manuscript
**Title and abstract**	1	(*a*) Indicate the study's design with a commonly used term in the title or the abstract	Title
		(*b*) Provide in the abstract an informative and balanced summary of what was done and what was found	Abstract (line 16 – 34)
**Introduction**			
Background/rationale	2	Explain the scientific background and rationale for the investigation being reported	Introduction (line 73 – 90)
Objectives	3	State specific objectives, including any prespecified hypotheses	Introduction (line 99 – 102)
**Methods**			
Study design	4	Present key elements of study design early in the paper	Section "Study design and sampling" (line 105 – 114)
Setting	5	Describe the setting, locations, and relevant dates, including periods of recruitment, exposure, follow-up, and data collection	Section "Study design and sampling" (line 109 – 114)
Participants	6	(*a*) Give the eligibility criteria, and the sources and methods of selection of participants	Section "Study design and sampling" (line 115 – 123)
Variables	7	Clearly define all outcomes, exposures, predictors, potential confounders, and effect modifiers. Give diagnostic criteria, if applicable	Section "Study Measurements" (line 131 – 167)
Data sources/ measurement	8*	For each variable of interest, give sources of data and details of methods of assessment (measurement). Describe comparability of assessment methods if there is more than one group	Section "Study Measurements" (line 131 – 167)
Bias	9	Describe any efforts to address potential sources of bias	Section "Study Measurements" (line 131 – 167) and Section "Data Analysis" (line 170 – 184)
Study size	10	Explain how the study size was arrived at	Section "Study design and

			sampling" (line 109 – 112)
Quantitative variables	11	Explain how quantitative variables were handled in the analyses. If applicable, describe which groupings were chosen and why	Section "Data Analysis" (line 172 – 184)
Statistical methods	12	(*a*) Describe all statistical methods, including those used to control for confounding	Section "Data Analysis" (line 172 – 186)
		(*b*) Describe any methods used to examine subgroups and interactions	Section "Data Analysis" (line 178 – 186)
		(*c*) Explain how missing data were addressed	Not applicable
		(*d*) If applicable, describe analytical methods taking account of sampling strategy	Not applicable
		(*e*) Describe any sensitivity analyses	Section "Data Analysis" (line 180 – 186)
**Results**			
Participants	13	(a) Report numbers of individuals at each stage of study—eg numbers potentially eligible, examined for eligibility, confirmed eligible, included in the study, completing follow-up, and analysed	Section "Study design and sampling" (line 105 – 114)
		(b) Give reasons for non-participation at each stage	Not applicable
		(c) Consider use of a flow diagram	Not applicable
Descriptive data	14	(a) Give characteristics of study participants (eg demographic, clinical, social) and information on exposures and potential confounders	Included in "Table 1" and "Table 2" (line 203 – 230)
		(b) Indicate number of participants with missing data for each variable of interest	Included in all tables
Outcome data	15	Report numbers of outcome events or summary measures	Section "Results" (line 207 – 226) and Tables 1 – 3.
Main results	16	(*a*) Give unadjusted estimates and, if applicable, confounder-adjusted estimates and their precision (eg, 95% confidence interval). Make clear which confounders were adjusted for and why they were included	Section "Results" (line 207 – 226) and Tables 1 – 3.
		(*b*) Report category boundaries when continuous variables were categorized	Section "Results" (line 207 – 226) and Tables 1 – 3.
		(*c*) If relevant, consider translating estimates of relative risk into absolute risk for a meaningful time period	Not applicable

Other analyses	17	Report other analyses done—eg analyses of subgroups and interactions, and sensitivity analyses	Section "Results" (line 245 – 266) and Tables 4.
**Discussion**			
Key results	18	Summarise key results with reference to study objectives	Line 270 – 283.
Limitations	19	Discuss limitations of the study, taking into account sources of potential bias or imprecision. Discuss both direction and magnitude of any potential bias	Line 280 – 405 and section "Strengths and limitations" (line 422 – 437)
Interpretation	20	Give a cautious overall interpretation of results considering objectives, limitations, multiplicity of analyses, results from similar studies, and other relevant evidence	Line 284 – 420.
Generalisability	21	Discuss the generalisability (external validity) of the study results	Line 284 – 420.
**Other information**			
Funding	22	Give the source of funding and the role of the funders for the present study and, if applicable, for the original study on which the present article is based	Section "Funding" (line 470 – 473)

Dietary Data

The following presents data on the diet of the analyzed sample, based on household responses (n: 414), in Table S1 and S2, for the surveys Kidmed and FIES, respectively.

Table S2. Responses to each question of the KidMed survey.

**Question**	**Yes**	**No**
Takes a fruit every day	303 (73,20%)	111 (26,80%)
Has a second fruit every day	161 (38,90%)	253 (61,10%)
Has fresh or cooked vegetables regularly once per day	297 (71,70%)	117 (28,30%)
Has fresh or cooked vegetables more than once per day	128 (30,90%)	286 (69,10%)
Consumes fish regularly (at least 2–3 times per week)	249 (60,10%)	163 (39,40%)
Goes to a fast-food (hamburger) restaurant more than once per week	107 (25,80%)	307 (74,20%)
Likes pulses and eats them more than once per week	292 (70,50%)	122 (29,50%)
Consumes whole-grain pasta or whole-grain rice almost every day (5 or more times per week)	212 (51,20%)	202 (48,80%)
Consumes nuts regularly (at least 2–3 times per week)	159 (38,40%)	255 (61,60%)
Uses olive oil at home	326 (78,70%)	88 (21,30%)
Have breakfast every day	364 (87,90%)	50 (12,10%)
Have cereal or grain-based foods (bread, toast, etc.) for breakfast	230 (55,60%)	184 (44,50%)

Has commercially baked goods or pastries for breakfast	143 (34,50%)	271 (65,50%)
Has a dairy product for breakfast (yoghurt, milk, etc.)	346 (83,60%)	68 (16,40%)
Takes two yoghurts and/or some cheese (40 g) daily	259 (62,60%)	155 (37,40%)
Takes sweets and candy several times every day	105 (25,40%)	309 (74,60%)

Table S3. Responses to each question of the FIES scale. At the interview, all the questions begin with - *During the last 12 months, was there a time when, because of lack of money or other resources…*

**Question**	**Yes**	**No**
You were worried you would not have enough food to eat?	179 (43,20%)	232 (56,00%)
You were unable to eat healthy and nutritious food?	102 (24,60%)	309 (74,60%)
You ate only a few kinds of foods?	105 (25,40%)	306 (73,90%)
You had to skip a meal?	15 (3,60%)	396 (95,70%)
You ate less than you thought you should?	21 (5,10%)	390 (94,20%)
Your household ran out of food?	10 (2,40%)	401 (96,90%)
You were hungry but did not eat?	6 (1,40%)	405 (97,80%)
You went without eating for a whole day?	3 (0,70%)	408 (98,60%)

Table S3 shows the linear correlation coefficient for each of the variables measuring the nutritional status of the students with the KidMed index, along with the adjusted linear regression model and the associated p-value for this relationship. The most significant relationships are found with weight (p-value = 0.005), BMI (p-value = 0.030), waist circumference (p-value = 0.032), hip circumference (p-value = 0.002), and arm circumference (p-value = 0.034). In this case, it can be observed that all significant relationships are negative, indicating that greater adherence to the Mediterranean Diet is associated with lower values of the nutritional status variables that are compatible with poorer weight status. In other words, based on the data, it could be said that higher adherence to the Mediterranean Diet, as measured by the KidMed index, may provide protection against overweight or obesity in this population.

Table S4. Relationship between nutritional status and the KidMed Index.

**Variable**	r	Beta	IC95%		p-valor
**Weight**	-0,140	-0,993	-1,688	-0,298	0,005
**BMI**	-0,109	-0,202	-0,384	-0,020	0,030
**Z-Score**	0,020	0,012	-0,048	0,072	0,690
**Waist**	-0,107	-0,506	-0,969	-0,043	0,032
**Hip**	-0,152	-0,884	-1,451	-0,317	0,002
**Waist-Hip Index**	0,088	0,003	0,000	0,007	0,080

**Waist-Height Index**	-0,004	0,000	-0,003	0,003	0,929
**Brachial Circumference**	-0,107	-0,205	-0,394	-0,016	0,034
**Tricipital Skinfold**	-0,075	-0,285	-0,658	0,088	0,133
